# Sex and gender-related differences in neurological diseases: current challenges and recommendations for clinical practice

**DOI:** 10.1007/s10072-025-08623-8

**Published:** 2026-01-06

**Authors:** Calogero Edoardo Cicero, Luca Angelini, Gianmarco Abbadessa, Matilde Bruno, Giulia Fiume, Bruna Nucera, Raffaele Ornello, Gennarina Arabia, Loretta Giuliano, Biancamaria Guarnieri, Alessandra Lugaresi, Daniela Perani, Simona Sacco, Cristina Tassorelli, Alessandra Nicoletti, Maria Teresa Pellecchia

**Affiliations:** 1 Department of Medical, Surgical and Advanced Technologie “G.F. Ingrassia”, University of Catania, Catania, Italy; 2 IRCCS Neuromed, Pozzilli, IS Italy; 3 Department of Advanced Medical and Surgical Sciences, University of Campania Luigi Vanvitelli, Naples, Italy; 4 Department of Brain Sciences, Imperial College London, London, UK; 5 Memory Clinic and Neurodegenerative Dementia Research Unit, University of Rome Tor Vergata, Rome, Italy; 6 UOC Neurologia, IRCCS Centro Neurolesi Bonino-Pulejo, Messina, Italy; 7 ⁠Department of Neurology, Hospital of Merano (SABES-ASDAA), Teaching Hospital of the Paracelsus, Medical Private University (PMU), Merano-Meran, Italy; 8 Paracelsus Medical University, Salzburg, 5020 Austria; 9 Department of Biotechnological and Applied Clinical Sciences, University of L’Aquila, L’Aquila, Italy; 10 Institute of Neurology, Department of Medical and Surgical Sciences, University “Magna Graecia”, Catanzaro, 88100 Italy; 11 Neurology Clinic, University Hospital G. Rodolico San Marco, Catania, Italy; 12 Department of Neurology Villa Serena Hospital Città Sant´ Angelo, Center of Sleep Medicine, Pescara, Italy; 13 Fondazione Villaserena per la ricerca, Città Sant´Angelo, Pescara, Italy; 14 Department of Biomedical and Neuromotor Sciences, University of Bologna, Bologna, Italy; 15 IRCCS Institute of Neurological Sciences, Bologna, Italy; 16 Vita-Salute San Raffaele University, Milan, Italy; 17 Department of Applied Clinical Sciences and Biotechnology, University of L’Aquila, L’Aquila, Italy; 18 Department of Brain and Behavioral Sciences, University of Pavia, Pavia, Italy; 19 IRCCS C. Mondino Foundation, Pavia, Italy; 20 Department of Medicine, Surgery and Dentistry “Scuola Medica Salernitana”, Neuroscience Section,, University of Salerno, Salerno, Italy; 21 Neurological Clinic, AOU San Giovanni di Dio e Ruggi d’Aragona, Salerno, Italy

**Keywords:** Sex-differences, Alzheimer’s disease, Parkinson’s disease, Epilepsy, Headache, Stroke, Multiple sclerosis

## Abstract

Neurological diseases include a large variety of conditions ranging from inflammatory, vascular and neurodegenerative disorders to epilepsy and headache. The impact of sex and gender on various aspects of these conditions (epidemiology, risk factors, pathophysiology, clinical features, treatment, and management of pregnancy and breastfeeding) is still not entirely taken into consideration, despite a rapidly increasing body of evidence. This position paper covers six neurological conditions (Alzheimer’s Disease, Cerebrovascular disease, Parkinson’s disease, Epilepsy, Headache disorders, Multiple Sclerosis) providing an overview of available evidence on sex and gender differences, identifying knowledge gaps and providing recommendations for clinical practice and future studies. We recommend taking into consideration modifiable sex and gender specific risk factors, the role of hormones across women’s lifespan and a personalized treatment approach based on gender. We also recommend that future efforts should be devoted to increase the representation of women in clinical studies, to promote sex and gender-based guideline production and to better characterize the safety profile in pregnancy of newer drugs.

## Introduction

Gender medicine is a field of medicine devoted to studying the impact of sex and gender (and their interaction) on different aspects of disease processes, such as epidemiology, risk modulation, clinical manifestations, and therapeutic response. Interest in this field stems from growing body of evidence showing that men and women frequently diverge in many disease aspects, impacting the perception of the disease and the way physicians’ approach and treat a patient. Neurological disorders encompass a wide range of conditions including inflammatory, vascular, and neurodegenerative diseases, which in turn are influenced by both sex and gender.

This rapidly growing interest has been demonstrated by the increasing number of publications dedicated to the subject. However, although the importance of sex inclusiveness in clinical research is well recognized, sex bias remains prevalent in biomedical research. In fact, despite the passage of National Institutes of Health (NIH) and United States Congress policies in 2015 and 2016 to increase women enrolment in clinical research [1], women are still under-represented in clinical trials and sex bias still exists with consequence in the external validity of the benefit/risk assessments of launched drugs. As a matter of fact, to date there is a lack of gender-oriented recommendations for therapeutic management of neurological diseases where gender and sex differences are recognized.

In order to provide a reference framework, this position paper aims (1) to provide an overview of available evidence on sex and gender differences that might have implications for clinical management of the most common neurological diseases, (2) to identify the most important knowledge gaps in the field and (3) to provide, wherever possible, recommendations for clinical practice and directions for future studies.

## Methods

A multidisciplinary panel from the Gender Neurology Study Group and the Young Members Section of the Italian Society of Neurology was involved in this position paper.

The overview of the existing literature was conducted by searching literature on the topic of sex and gender and six neurological conditions (Alzheimer’s disease, Cerebrovascular diseases, Parkinson’s Disease, Headache disorders, Epilepsy and Multiple Sclerosis). All the relevant papers were selected, including systematic reviews, editorials and point of view. From each paper, information was gathered on gender differences in the epidemiology, risk factors and pathophysiology, clinical features, management of pregnancy and breastfeeding where applicable and therapeutic approach of a specific neurological condition. The evidence was used by the panel members to support their recommendations and suggest strategies for future studies for each neurological condition.

### Alzheimer’s disease

#### Epidemiology

By 2050, the global number of people living with dementia is expected to overtake 150 million—tripling the estimated 50 million cases in 2019, with a significant contribution from low and middle income, where incidence rates are expected to increase [2]. Among all the causes of dementia, Alzheimer’s disease (AD) stands out as the most prevalent, with a female-to-male ratio of 1.69 (1.64–1.73) [2]. Incidence rates are generally higher for women, and significantly increase after the age of 80 years in high income countries [3]. Moreover, when considering death caused by dementia, the rates are significantly higher for women compared to men, even adjusting for the higher life expectancy (and thus increased risk of dementia) in women [4].

#### Risk factors and pathophysiology

Among the risk factors for sporadic AD, age remains the one exhibiting the most significant sex and gender difference. The primary reason offered for this discrepancy is women’s greater longevity [5]. However, some age-controlled studies did not show this association or suggest that sex differences in incidence rates only emerged after the age of 80 [6].

In addition to age, the Apolipoprotein (APOE) ε4 allele is the strongest known genetic risk factor for late-onset AD. Its impact is not uniform across sexes, with growing evidence showing it interacts differently in men and women. Women show higher tau accumulation and stronger amyloid–tau links, while men experience more neurodegeneration and cognitive decline [7]. Women with high tau levels show faster cognitive decline, and women APOE ε4 carriers with amyloid positivity face a higher risk of decline than men [8, 9].

The 2020 Lancet Commission identified twelve modifiable risk factors for dementia, including both biological and socioeconomic elements: low education/occupation, hypertension, hearing loss, smoking, midlife obesity, depression, physical inactivity, diabetes, social isolation, excessive alcohol use, traumatic brain injury, and air pollution. In 2024, untreated vision loss and high LDL cholesterol were added [10]. All known modifiable risk and protective factors show sex/gender differences in prevalence or impact [11]. For example, women with type 2 diabetes experience faster cognitive decline [12]. Declining 17β-estradiol during menopause adds further risk in women [12], along with increased obesity, insulin resistance, hypertension, and dyslipidemia—all factors linked to higher AD risk in women [13].

In the last decades, an increasing body of literature demonstrated that sleep and circadian alterations are risk factors for AD, with a need to balance different risk profiles in men and women. Some apparent contradictions have emerged: insomnia- highly prevalent in women since middle age- and OSA- highly prevalent in men- are both risk factors for AD [14]. It is noteworthy that menopause increases OSA prevalence in women, with more detrimental cognitive effects even in less severe OSA forms [15]. Sleep and circadian alterations, more prevalent in the menopausal period [16], interfere with amyloid and tau spread and deposition from the preclinical phases of AD through a vicious cycle involving local sleep aspects, sleep fragmentation leading to impairment of the glymphatic system, cerebrovascular dysfunction, dysregulation of orexinergic system, neuronal and astrocytic activity [17].

Beyond these biological mechanisms, sex and gender also shape the social and emotional dimensions of AD. Potentially modifiable lifestyle factors such as education, occupation, social activities, exercise, and cultural factors appear to play important roles in the increased risk of AD in women and may help explain both gender and geographical differences in risk of the disease.

Socioeconomic factors impact also caregivers of AD patients, of which two-thirds are represented by women [18]. Caring for a spouse with dementia is associated with a 30% increase in depressive symptoms [19], and is burdened by more substantial emotional, financial and physical difficulties compared to caregivers of subjects without dementia [20].

Men and women show discrepancies in neuropathology and neurodegeneration cascade processes. In a recent study, there was no significant sex difference in baseline Aβ load in AD patients, but females displayed a more widespread tau pathology at follow-up, in line with previous data [21, 22]. Men and women respond differently to Aβ deposition, with a synergistic interaction between Aβ and p-tau, contributing to faster cognitive decline and dementia progression in women. Notably, a higher cerebrospinal fluid (CSF) p-tau181 concentration is found in women [22], suggesting a sex-specific modulation of tau phosphorylation in response to Aβ [23].

Findings from autopsy reports [24], neuroimaging [25], and CSF analyses [26], consistently showed elevated levels of pathological tau in women —both those diagnosed with AD and those at increased risk due to the presence of APOE4 or significant Aβ accumulation in the brain. Given the strong association between tau distribution and clinical symptoms [27], the elevated tau burden in women may help explain both the higher prevalence and more severe progression in women AD patients.

Differently from the aforementioned biomarkers, consistent data are currently lacking regarding sex-related differences in the novel plasma biomarkers [28].

Emerging evidence indicates non-neuronal contributions to AD pathogenesis, such as neuroinflammation, emphasizing the importance of extending sex-related investigations to this aspect. In AD mouse models, female microglia transition into activated states more rapidly and exhibit heightened inflammatory gene expression in response to amyloid pathology [29]. Notably, a sex-biased efficiency in amyloid plaque coverage has been observed, with female sex and APOE4 genotype jointly associated with reduced microglial plaque coverage and increased amyloid burden [30]. The inflammatory responses are also modulated by estrogens, which fulfill multiple functions, from the mitochondrial respiratory chain to the maintenance of synaptic integrity and function in animal models [31].

Some individuals resist dementia progression despite AD pathology—a phenomenon explained by the cognitive reserve (CR) and brain reserve (BR) hypothesis, which defines CR as the influential effect on brain’s adaptive capacity to maintain function despite aging or damage [32]. Both biological and socio-economic factors, related to sex and gender differences, influence CR and BR, and represent active research areas. Education, a common CR proxy, is linked to brain health and connectivity [33]. A 2024 study confirmed that a high education helps counteract cognitive decline; in low-education groups—where more women were represented and fewer had occupational experience—low literacy, diabetes, and long sleep were negative factors, while cognitive and physical activity were beneficial [34]. Perneczky et al. [35] showed that sex modulates CR effects on brain metabolism. Men exhibit changes in posterior associative regions (precuneus, temporo-parietal cortex), while women in anterior areas (medial frontal, orbitofrontal cortex), and limbic structures [36]. These results underline the potential neural basis of these brain gender differences likely related to modifiable sociodemographic factors.

#### Clinical features

Men and women differ in the clinical manifestation of the disease, with women scoring better in tasks evaluating attention, verbal and face memory, and social cognition, and men in sensorimotor speed and spatial orientation [37]. Although women report better performances in memory tests at baseline, they exhibit an accelerated cognitive decline along the disease course, particularly in episodic memory, resulting in worse longitudinal progression [38, 39].

In addition, female sex is associated with more frequent and progressively severe neuropsychiatric symptoms [40]. In particular, women with AD dementia show higher prevalence and severity of depressive symptoms, aberrant motor behavior, and psychotic symptoms, while men show increased severity of apathy [41]. The interindividual differences in the manifestation of neuropsychiatric symptoms stem from neurobiological mechanisms, medical conditions, and possibly cultural factors including gender roles. In addition, these aspects can influence correct diagnosis delaying a timely taking in charge of the disease in women [42].

#### Treatment

Compared to men, women often experience delays in receiving diagnoses and are more likely to undergo inappropriate interventions. These disparities are influenced by systemic biases within healthcare, including gender stereotypes, benevolent sexism, and ageism [43]. Healthcare professionals may unconsciously subscribe to notions that portray women as more emotional and less rational, thereby affecting clinical judgments regarding diagnosis and treatment [5]. Additionally, prevailing perceptions that women require protection and care can lead to patronizing attitudes and decisions that undermine their autonomy and perceived competence [44].

Setting aside the different approaches healthcare professionals may pursue, there is evidence that treatment responses for AD vary by sex as well. Cholinesterase inhibitors are the most widely examined therapy [45]. Some studies report that men experience stronger benefits, while others note that women are more likely to respond to donepezil and rivastigmine despite a greater risk for some side effects, such as bradycardia and adverse reactions to sedative-hypnotics [46]. New treatments also show distinct sex-based patterns. Lecanemab appears to confer greater benefits in men, with one study reporting significant differences in efficacy, with a potentially limited effect in women [47]. A summary of available evidence and knowledge gaps are presented in Table 1.

**Table 1:** Main sex/gender differences in alzheimer’s disease and knowledge gaps

Topic	Summary of available Evidence	Knowledge gaps
Epidemiology	Women show a higher prevalence of AD (F: M ratio 1.69), partly due to longevity. Sex differences emerge more clearly after age 80.	Limited understanding of whether higher women prevalence is due solely to longevity or other sex-specific mechanisms.
Risk factors	Sex/gender differences influence age, APOE ε4 effect, comorbidities (e.g., diabetes, obesity), sleep disturbances, and socioeconomic factors. Women show greater biological and socio-cultural vulnerability. Menopause and female hormonal changes during lifetime play a pivotal role.	Insufficient sex and gender stratified data on modifiable risk factors and the interaction between biological and socio-cultural determinants.
Clinical presentation	Women perform better on verbal memory at baseline but decline faster. They exhibit more neuropsychiatric symptoms (depression, psychosis), while men show more apathy.	Need for better characterization of sex/gender-specific trajectories and neuropsychiatric symptom patterns in AD.
Pathophysiology	Women exhibit greater tau pathology and a stronger Aβ–p-tau interaction. Sex differences in neuroinflammation and microglial response are influenced by estrogens.	Lack of consistent data on sex differences in novel biomarkers (e.g., plasma markers) and neuroinflammation.
Cognitive reserve and resilience	Sex/gender-related factors such as education and socioeconomic status modulate cognitive and brain reserve. Women with lower education show higher vulnerability.	Limited research on how brain networks involved in cognitive reserve differ between sexes and genders in structure, function, and compensatory strategies.
Treatment	Women experience diagnostic delays and more inappropriate treatments. There are sex differences in response and side effects to both traditional and novel anti-amyloid therapies.	Underrepresentation of sex-specific analyses in trials; unclear differential responses to new AD therapies.

#### Recommendations and future directions

- Consider sex and gender as key determinants of interindividual differences in the risk of progression, neuropathology, clinical and biological presentation, and treatment response of AD. Tailored, sex/gender -informed approaches to deliver individualized care to patients with AD and their caregivers are needed.
- Promote development of comprehensive and rigorous guidelines to ensure that sex and gender are appropriately integrated into the analysis and reporting of clinical study data, as well as in the diagnosis and treatment of AD. Such efforts could pave the way for more inclusive and targeted intervention strategies, and transformative policies that may enhance our ability to diagnose, treat, and prevent AD [48].
- Sustain public health interventions targeting modifiable risk factors, that offer a promising avenue for lowering disease prevalence [2].

### Cerebrovascular disease

#### Epidemiology

Cerebrovascular disease includes ischemic stroke (IS), intracerebral hemorrhage (ICH), subarachnoid hemorrhage (SAH), and cerebral venous thrombosis (CVT). The World Stroke Organization published in 2025 the Global Stroke Fact Sheet [49] which includes analyses of stroke burden derived from The Global Burden of Disease 2021 Study on stroke. According to this data, each year about 47% of all strokes occurs in women; additionally, 49% of people who live with a previous stroke are women.

By subtype, IS in women has a crude incidence rate of 96 [95% UI 83–110] per 100,000 persons per year, a prevalence of 50% and a mortality of 50%. ICH in women has a crude incidence rate of 39 [95% UI 34–43] per 100,000 persons per year, a prevalence of 44% and a mortality of 45%. SAH in women has a crude incidence rate of 9 [95% UI 8–10] per 100,000 persons per year, a prevalence of 55% and a mortality of 51% [50].

Considering age, the incidence of stroke in women remains lower than men up to 75 years, after which it reverses. This gap is present in both IS and ICH [51]. In SAH, however, there is a higher incidence rate in women over 50 years [52]. Also, in CVT there is a higher incidence rate in women, especially in an age group between 31 and 50 years [53, 54].

The lifetime risk of stroke is generally higher in men. For women, the countries with the highest risk are Eastern Europe (36.5%) and East Asia (36.3%) [50].

#### Risk factors and pathophysiology

Recent studies have observed that the heritability of IS is higher in women than in men [55]. The impact of common risk factors on women is variable. Stroke occurs at an older age in women compared to men, and this is accompanied by higher rates of hypertension and atrial fibrillation (AF) [56].

Some risk factors common to both sexes seem to have a greater impact on women. For example, arterial hypertension appears to have a greater effect on the risk of stroke in women [57]; AF is associated with double the risk of stroke in women [58]; diabetes mellitus is less common in women but is associated with greater severity of stroke [59]. In particular, AF has been recognized as a sex-specific risk factor and female sex has therefore been included in the CHA₂DS₂-VASc score to compute for the increased risk. Additionally, age appears to modify the association between sex and stroke risk in patients with AF, with the risk becoming higher in women after the age of 80 [60].

It is well known that women are protected from stroke risk during their fertile period, although the mechanisms underlying this phenomenon are not yet fully understood. Since women’s risk for cardiovascular disease increases after menopause, it is possible that female sex hormones play a protective role against atherosclerosis and vascular diseases in general [61]. On the other hand, pregnancy is a phase of life characterized by significant physiological changes, including an increased risk of stroke, particularly in the third trimester and the postpartum period. This is due to venous stasis, which leads to greater edema and fluid retention, as well as a hypercoagulable state caused by reduced levels of protein S, increased fibrinogen levels, and resistance to activated protein C [62]. Exogenous hormones also play a significant role. Combined estrogen-progestin contraceptives are associated with an increased risk of ischemic stroke, which is directly related to the hormone dose [63], especially in thrombophilic patients [64]. Furthermore, postmenopausal hormone replacement therapy with estrogens alone or in combination with progestins also increases the risk of stroke [65].

It is also possible to identify sex-specific risk factors for cerebrovascular diseases: early menarche and menopause, pregnancy and complications (gestational hypertension and related complications, gestational diabetes, etc.), use of hormonal contraceptives, and hormone replacement therapy [66]. A meta-analysis of 24 studies found that combined oral contraceptive (COC) use increases the risk of myocardial infarction or ischemic stroke by 1.6 times, with the highest risk linked to pills containing more than 50 µg of estrogen [67]. In a large cohort study, the use of combined hormonal contraceptives (CHCs) was associated with a 1.77 times higher incidence rate ratio (IRR) of ischemic stroke compared to nonuse. Notably, CHCs with fourth-generation progestins were associated with a 30% lower IRR compared to second-generation CHCs, while third-generation progestins showed no significant difference from that of second-generation users [68]. One common risk factor, potentially present both in men and women but with greater impact in women, is migraine with aura, especially if aged < 45 years. The impact of migraine with aura on the risk of IS is magnified in younger women, especially if they are smokers and are on estroprogestinic treatment [69].

Regarding the etiology of ICH, no differences were observed between the sexes. The frequency of hypertensive hemorrhage and cerebral amyloid angiopathy (CAA) was similar in men and women [70].

The risk of ICH in women is partly explained by the fact that certain risk factors, which are common to both sexes, tend to have a stronger impact on women. For example, arterial hypertension tends to be more severe in women after the age of 30, due to hormonal changes associated with pregnancy, the postpartum period, and conditions such as polycystic ovary syndrome [71]. Menopause is considered a specific risk period for cerebral hemorrhage, as it is associated with a reduction in estrogen levels, increased blood pressure, alterations in the lipid profile, and increased insulin resistance [72].

SAH also shows sex-specific patterns. There is higher prevalence of cerebral aneurysms in women than in men [73]. However, although the risk of rupture is higher in women than in men [74], this difference is not supported by characteristics related to aneurysm features or women’s risk factors [75]. It appears that there are differences in the hemodynamics of the women cerebral circulation [70], as well as anatomical differences in aneurysm distribution (e.g., a higher incidence at the level of the posterior communicating artery) [71].

Finally, CVT is associated with hormonal risk factors that contribute to its higher prevalence in women. In particular, the use of hormonal contraceptives, especially in women with an underlying prothrombotic condition, increases the risk of CVT. Pregnancy itself also represents a temporary risk factor for CVT [76].

#### Clinical features

Several studies on the clinical characteristics of ischemic and hemorrhagic stroke have not found significant differences between the sexes [77, 78]. However, some research suggests that although the most common initial symptoms are similar in men and women, women are more likely than men to present with non-focal symptoms, which can increase the risk of misdiagnosis. These often include non-focal neurological signs such as generalized fatigue, confusion, nausea, vomiting, headache, and memory disturbances [79, 80].

Women also seem to have a higher incidence of ischemic strokes involving the anterior circulation and with cardioembolic origin, which may be attributed to the greater prevalence of atrial fibrillation in women [81]. Data on the higher prevalence of collateral circulation by gender and its association with better outcomes are conflicting [82]. In the case of intracerebral hemorrhage (ICH), few radiological studies directly compare men and women. Some studies suggest that women may have smaller hematoma volumes and a perilesional edema [83], but larger studies are needed to confirm these findings.

Women tend to have higher stroke severity as indicated by the National Institutes of Health Stroke Scale (NIHSS) scores at admission compared to men [84], and a greater likelihood of residual disability between one and ten years after the ischemic event [85]. This may be because women often experience stroke at an older age and with more comorbid conditions, or because they are more likely to arrive at the hospital later, due to the prevalence of non-specific symptoms [86].

Overall outcomes, in terms of mortality, are worse in women than in men from the age of 75 onward. Women also exhibit higher rates of post-stroke anxiety and depression, as well as post-stroke pain and disability [65]. This appears to be partly explained by older age, a greater burden of comorbidities, and reduced social support [87].

#### Treatment

In cases of IS, acute-phase reperfusion therapies, intravenous thrombolysis (IVT) and mechanical thrombectomy (MT) have shown comparable results in clinical trials in terms of 90-day mortality, functional independence (assessed using the modified Rankin Scale, mRS), safety, and recanalization rates between genders [88, 89]. Thus, in clinical practice, women should receive the same treatments of men in the setting of acute IS. However, long-term outcomes appear to be worse in women, particularly following MT. This may be due to the fact that women enrolled in trials are often older, have lower levels of pre-stroke functional independence, and present with more severe comorbidities compared to men [90]. Therefore, future studies should consider adjusting for age and baseline functional status. Furthermore, women appear to be less frequently eligible for intravenous thrombolysis (IVT) compared to men. This could be attributed to the higher prevalence of atrial fibrillation in women, which makes them more likely to be on anticoagulant therapy at the time of the ischemic event—an absolute contraindication to IVT [91]. Additionally, the fact that women more often live alone and frequently present with atypical symptoms can contribute to delays in stroke recognition and hospital arrival [92].

For primary and secondary stroke prevention, no gender specific recommendations regarding common medical treatments such as antithrombotics, antihypertensive, lipid-lowering drugs and antidiabetics are available. [93–96], 

Several consensus statements advise against the use of estrogen-progestin therapy in women with migraine with aura due to the associated increased risk of stroke [97].

The treatment of ICH and SAH is similar between men and women. However, it is important to note that women are less frequently treated in stroke units and receive less intensive care overall, which may contribute to their worse 3-month outcomes compared to men [98, 99].

#### Pregnancy and breastfeeding

Pregnant women have a threefold higher risk of ischemic stroke, intracerebral hemorrhage, and cerebral venous thrombosis compared to non-pregnant women of the same age [100]. Some pathological conditions that occur more frequently during pregnancy and can lead to stroke should be mentioned. One such condition is posterior reversible encephalopathy syndrome (PRES), a form of hypertensive encephalopathy characterized by reversible cerebral edema, which may be complicated by IS or ICH [101]. Another one is reversible cerebral vasoconstriction syndrome (RCVS) that can lead to IS [102]. Both conditions are commonly observed in women with hypertensive disorders of pregnancy, including gestational hypertension, preeclampsia, eclampsia, and HELLP syndrome (Hemolysis, Elevated Liver enzymes, and Low Platelets).

Treatment of acute IS in pregnancy poses peculiar challenges and there is adjunctive concern to preserve pregnancy and avoid potential harms to the fetus. Pregnancy is an exclusion criterion for all clinical trials and thus our knowledge on benefits and harm of potential treatments is based on case series and registry data. Alteplase and Tenecteplase are large molecules that do not cross the placental barrier and, according to animal studies, are not teratogenic at the doses used for revascularization therapy [103].

The European Stroke Organization guideline is currently unable to provide evidence-based recommendations regarding IVT in pregnant women due to lack of data from clinical trials. However, based on observational data, most experts suggested treating pregnant women with disabling ischemic stroke with IVT. A similar recommendation applies to women who aren’t in the immediate postpartum period. As for treatment of acute IS with MT, although data are again limited to case reports, the European Stroke Organization expert consensus statement recommends considering MT in acute IS with large vessel occlusion. In such scenarios, MT alone is generally preferred over IVT or bridging therapy [104]. Nevertheless, it’s crucial to include pregnant women in clinical studies in order to establish clearer treatment indications. In this regard, the prospective, observational, international, multicenter SiPP (Stroke in Pregnancy and Postpartum) study has already been initiated to investigate the pathophysiology, clinical features, treatment, and outcomes of cerebrovascular events during pregnancy and the postpartum period [96].

In cases of ICH or SAH, the management of arterial hypertension is essential. During pregnancy, antihypertensive treatment should be initiated gradually, starting with the drugs that are safest for both the mother and the fetus, and adjusted based on clinical response [105]. The use of nimodipine is considered safe during pregnancy and should be used when indicated for the prevention of vasospasm—especially considering its established use in the management of preeclampsia [106]. It’s also crucial to identify the underlying cause and initiate appropriate treatment promptly. The literature reports cases of aneurysm treatment by coiling or clipping [107].

In the presence of intracranial hypertension, in pregnancy, the use of hyperosmolar therapy with mannitol or hypertonic saline is still not supported by clinical studies in humans. Mannitol is known to cross the fetoplacental barrier and is excreted in fetal urine. Animal studies suggest that the osmotic effects of mannitol can lead to fetal dehydration, with decreased oxygenation, reduced plasma volume, and disturbances in acid–base balance [108].

Finally, in cases of CVT, the use of low molecular weight heparin at therapeutic doses is considered safe during pregnancy [109], and should be continued for at least six weeks postpartum [76]. No data are available regarding the effects of these treatments during breastfeeding.

A summary of available evidence and knowledge gaps are presented in Table 2.

**Table 2:** Main sex/gender differences in cerebrovascular diseases and knowledge gaps

Topic	Summary of available evidence	Knowledge gaps
Epidemiology	Stroke is less common in women than in men, but its severity is generally greater in women. Pregnant women have a threefold increased risk of IS, ICH and CVT compared to age-matched non-pregnant women.	There is limited epidemiological data on women in developing countries, especially on pregnant women.
Pathophysiology	Women exhibit sex-specific and age-related stroke risk factors, including hormonal influences, pregnancy-related conditions, and increased sensitivity to common risk factors such as hypertension and atrial fibrillation. These factors contribute to greater stroke severity and distinct cerebrovascular disease profiles compared to men.	The mechanism by which hormones influence the pathophysiology of cerebrovascular events is not yet fully understood.
Clinical presentation	Women more often present with non-focal symptoms, exhibit greater stroke severity, and have worse functional outcomes, partly due to older age, comorbidities, and delayed presentation.	Data on collateral circulation in the setting of acute ischemic stroke are conflicting. Limited data on differences in ICH between women and men.
Treatment	Despite similar efficacy of acute-phase treatments for ischemic stroke between sexes, women often experience worse long-term outcomes—likely due to older age, greater comorbidity burden, lower pre-stroke independence, reduced eligibility for IVT, and delays in care.	No evidence-based recommendation about revascularization treatments during pregnancy. Limited data about treatments during breastfeeding.

#### Recommendations and future directions

- Consider that the incidence and prevalence of stroke is lower in women than in men, but the severity of stroke is generally worse in women.
- Consider sex-specific risk factors including migraine with aura, use of estroprogestinic, pregnancy and puerperium that warrant peculiar attention and consideration.
- Consider that at stroke onset women more frequently present with non-focal neurological symptoms, which may lead to misdiagnosis.
- Consider that stroke severity and outcomes tend to be worse in women because of differences in baseline risk factors and delays in diagnosis.
- Consider sex-specific risk factors for both primary and secondary prevention.
- Recognize specific pathological conditions that may arise during pregnancy in order to prevent acute cerebrovascular events.
- Promote clinical trials on acute revascularization treatments and other acute-phase therapies during pregnancy and postpartum, to increase evidence on efficacy and safety of such treatments in this population.

### Parkinson’s disease

#### Epidemiology

Parkinson’s disease (PD) is the second most common neurodegenerative disorder and is clinically characterized by bradykinesia, rigidity, rest tremor, and several non-motor symptoms.

Epidemiological studies consistently report a higher incidence and prevalence of PD in men compared to women. Among individuals aged 60–79, the incidence is approximately 1.6 times higher in men, and this ratio appears to be increasing over time [110, 111]. Although the prevalence remains higher in men [112], a recent meta-analysis suggests a potential trend toward sex convergence over time [113]. Data on sex differences in the age at disease onset are conflicting: while some studies report a later onset in women, others find no significant difference between sexes [114].

#### Risk factors and pathophysiology

The predominance of men in PD may be partially explained by sex-specific differences in exposure to environmental risk factors and in biological susceptibility. For instance, men may be more likely to work in occupations involving exposure to neurotoxic agents such as pesticides, solvents, and metals, which are associated with increased PD risk [115]. In terms of endogenous factors, high serum urate levels have been linked to a reduced risk of PD in both sexes, although the protective effect appears stronger in men, possibly due to hormonal interactions [116, 117]. Similarly, caffeine consumption is associated with a lower PD risk, particularly in men, and this effect may be modulated by estrogen [114, 118]. Physical activity also shows a more pronounced protective effect in men [119]. The relationship between female reproductive factors and PD risk remains unclear. Some studies suggest that later menopause and longer fertility may confer protection, while hormone replacement therapy and oral contraceptives have been associated with increased risk [120, 121]. However, other studies have found no significant associations [122]. Likewise, the contribution of head trauma to PD risk shows inconsistent sex-specific findings [114]. Regarding sex differences in genetic risk factors for PD, a higher proportion of women has been observed among North American and European patients with GBA mutations, as well as among those carrying the LRRK2 G2019S mutation, suggesting these variants may be more prevalent in women [123, 124]. However, a recent GWAS meta-analysis found no significant genetic differences between sexes [125].

Sex-related differences in the PD pathophysiology likely arise from complex interactions between sex hormones, gene regulation, and environmental factors. Estrogens have been shown to exert neuroprotective effects through several molecular pathways, and sex-specific gene expression and epigenetic patterns have been observed in PD-relevant brain regions such as the substantia nigra [126]. These differences also extend to neuroinflammatory responses: men exhibit a more pro-inflammatory profile in both serum and brain, and animal studies confirm greater neuroinflammation and dopaminergic vulnerability in men, effects likely influenced by sex hormones [127]. In addition, recent evidence suggests that sex-related differences in post-transcriptional regulation, including microRNA expression, may further contribute to divergent disease mechanisms and progression [128].

#### Clinical features

PD diagnosis relies on cardinal motor symptoms, and growing evidence suggests that sex-related differences may influence clinical presentation and progression. Evidence show that women tend to have a later motor symptom onset, more frequently present with a tremor-dominant phenotype, and show higher striatal dopaminergic activity at diagnosis [114]. Furthermore, recent longitudinal data showed that men experience faster motor progression and require higher dopaminergic doses [129]. Although PD progression appears slower in women, they are more prone to develop certain motor complications such as levodopa-induced dyskinesia and wearing-off [130–132]. Sex-related differences in pharmacokinetic and estrogens may underlie these complications [133–135].

Regarding non-motor symptoms, female sex is associated with a higher prevalence of anxiety, depression, fatigue and psychotic symptoms, and more severe anxiety, depression, and fatigue, while male sex was associated with a higher prevalence of apathy, impulse control disorders, REM sleep behavior disorder, hypersomnolence, and suicide. Moreover, men seem more likely to experience urinary and sexual dysfunction [136–140]. Literature on gender-differences in cognitive impairment shows mixed results, but a number of longitudinal studies and a meta-analysis support a higher risk of MCI and dementia in men [141–143]. Greater non-motor fluctuations are reported in women, particularly in anxiety, mood, and pain [129, 144].

#### Treatment

Levodopa is the gold standard therapeutical option for PD. However, since its introduction in the therapeutic regimen, there is evidence of differences in the pharmacokinetics profile, with women showing higher bioavailability compared to men [133, 145]. It has been previously hypothesized that the differences in the pharmacokinetics of levodopa might be due to the different body weight between men and women, with the latter presenting higher levodopa levels, thus being more at risk of developing adverse effects [146]. However, subsequent studies demonstrated that sex is the main predictor of levodopa bioavailability, with women presenting levodopa AUC and Cmax, corrected by body weight, almost threefold higher than men at their first levodopa intake and a levodopa AUC 27% higher in women compared to men patients under chronic treatment [133, 145]. Short tandem repeat (CA_n_-STR) polymorphism in the dopamine receptor D2 gene may have a protective effect on dyskinesia development in men, while the sex–related risk of dyskinesia in women may be so strong that it overcomes any protective effect due to genetic factors [147]. In a recent prospective multicenter study aiming to assess the development of motor/nonmotor fluctuations and dyskinesia based on gender over a 2-year observation period in two hundred and sixteen patients (139 men, 77 women) PD patients starting levodopa, female gender was the strongest predictor of wearing off (Odds ratio [OR] = 1.930; *P* = 0.0333) and dyskinesia (OR = 3.405; *P* = 0.0228) after 2-year intake of levodopa [148]. Concerning other antiparkinsonian drugs, the evidence is poor. For the I-MAO inhibitors, two recent studies demonstrated no differences in terms of efficacy or adverse events between men and women treated with safinamide [149, 150].

In advanced PD, several therapeutic options are now available, including surgical therapies and infusional therapies. Deep Brain Stimulation (DBS) is one of the most established surgical therapies for PD, with significant improvement on the motor symptoms and quality of life. Studies that have demonstrated gender differences in response to subthalamic nucleus stimulation are few, with women experiencing a lower degree of response in terms of bradykinesia compared to men [151], a transient higher improvement in UPDRS score than men [152], or a transient lower response to DBS compared to men, with overlapping long-term outcomes [153, 154]. On the contrary, other studies found no differences in the motor outcomes [155, 156]. When investigating quality of life, evidence is poor with a study demonstrating a better quality of life in women undergoing DBS [157], whereas a recent investigation did not find any difference in quality of life between genders [158]. Of relevance, there seems to be a difference also in the access to DBS with women being less represented in DBS cohorts [154, 159].

No evidence is available for other advanced therapies such as MRgFUS and infusional therapies, despite the evidence that women are less likely to be referred for advanced therapies compared to men [160]. Moreover, a recent analysis of clinical trials for advanced infusional therapies found that women were consistently underrepresented [161].

#### Pregnancy and breastfeeding

PD is usually a disease of the advanced age, as such very few data are available concerning pregnancy and breastfeeding. However, the increasing maternal age at first pregnancy might lead more women to have a pregnancy after a diagnosis of PD. A recent literature review gathered information on 148 pregnancies of PD women while undergoing levodopa therapy, of whom 109 had data on fetal outcomes with the majority experiencing live births, 10 spontaneous abortion and 3 congenital abnormalities, data that are comparable to the general population [162]. A recent case series on 12 pregnancies, of whom one was conducted while on pramipexole therapy and one while on levodopa/carbidopa reported overall good pregnancy outcomes, albeit with the report of motor worsening in patients not taking dopaminergic drugs [163]. Little is known also concerning breastfeeding, with only one study showing low concentrations of levodopa in breastmilk [164]. Interestingly, one study described three women who underwent DBS before pregnancy and had good pregnancy outcomes, however two of them interrupted dopaminergic therapy fearing a possible impact on pregnancy [165].

A summary of available evidence and knowledge gaps are presented in Table 3.

**Table 3:** Main sex/gender differences in Parkinson’s disease and knowledge gaps

Topic	Summary of available evidence	Knowledge gaps
Epidemiology	PD is more prevalent and incident in men, especially in older age. Women may have later onset, but findings are inconsistent.	Conflicting data on age at onset and unclear trend toward sex convergence over time.
Risk Factors & Pathophysiology	Men are more exposed to environmental risks (e.g., pesticides); protective effects of urate, caffeine, and physical activity are stronger in men. Estrogens may have neuroprotective effects.	Limited understanding of hormonal and reproductive influences. Inconsistent data on genetic risk and sex-related mechanisms.
Clinical Features	Women more often have tremor-dominant PD, later onset, slower progression, and higher frequency of non-motor fluctuations. Men show faster motor decline and more cognitive impairment.	Mixed results on the presentation and differential burden of non-motor symptoms in men and women.
Treatment	Women show higher levodopa bioavailability and are more prone to dyskinesias and motor complications. Women are less likely to undergo DBS or infusion therapies.	Lack of sex-specific dosing recommendations. Limited evidence on antiparkinsonian drugs beyond levodopa. Sparse data on sex-related differences in response to and access to advanced therapies. Women remain underrepresented in clinical trials involving advanced treatments.
Pregnancy & breastfeeding	Most pregnancies in PD women on dopaminergic therapy have favorable outcomes.	Very limited evidence on medication safety during pregnancy and breastfeeding. No specific guidelines currently available.

#### Recommendations and future directions

- Consider that gender influences all aspects of PD, from risk factors to clinical presentation and response to therapy.
- Recognize and manage non-motor symptoms and non-motor fluctuations according to their different prevalence and severity in men and women affected by PD.
- Consider reducing levodopa dosage in women as compared to men since the early disease phase, in order to reduce motor complications.
- Improve referral and access to advanced therapies (DBS, infusional therapies, etc.) for women affected by PD.
- Promote research on the safety of antiparkinsonian drugs during pregnancy and breastfeeding.
- Consider post-hoc analysis of previously published clinical trials or meta-analysis of data already generated to increase knowledge of sex differences in efficacy and safety of antiparkinsonian treatments.
- Developers should consider including sex in clinical trial design, ensuring a proper stratification of men and women into experimental groups and pre-specified ad-hoc analyses of sex subgroups for both efficacy and safety of the drug under development.

### Epilepsy

#### Epidemiology and risk factors

According to the Global Burden of Disease (GBD) [166] in 2021, there were 51.7 million people with epilepsy (24 million with idiopathic epilepsy and 28 million with secondary epilepsy), with the bulk of the burden (> 80%) residing in low-income to middle-income countries. The global age-standardized prevalence rate was 658/100,000 (307/100,000 for idiopathic epilepsy and 350/100,000 for secondary epilepsy). The global age-standardized prevalence in 2021 was not substantially different between men and women (322/100,000 for men and 293/100,000 for women). There were no substantial sex differences in the incidence rates of idiopathic epilepsy in 2021, but death rates in men (2.1) were substantially greater than in women (1.4) [166]. Nonetheless various studies suggest that incidence and prevalence of epilepsy are slightly higher in men than women [167–169].

This difference could be attributed to a higher exposure of men to the most frequent risk factors for structural epilepsy and acute symptomatic seizures, such as trauma, stroke and infections. Possibly for the same reasons, also status epilepticus seems to be more prevalent in men [170–172]. On the other hand, Idiopathic Generalized Epilepsies (IGE) are notably more common in women with a female to male ratio of approximately 1.5 in various studies [173].

Sex differences in epilepsy have been described also in terms of seizure propensity and response to therapy. Different factors could be implicated such as body weight, steroid hormones, cytochrome P450 activity, neurotransmitters and biological differences in neuronal networks [174, 175].

#### Clinical features

Few studies assessed sex differences in clinical presentation of epilepsy and seizures.

In particular, in temporal lobe epilepsy (TLE), focal aware seizures seem to be more common in women while bilateral evolution appears to be more frequent in men [176, 177]. A retrospective observational study revealed significant differences between genders in the frequency of atonic seizures, which were more common in men with generalized epilepsy, and autonomic, visual, and psychic symptoms associated with focal epilepsy, which were more common in women [178].

Moreover, in TLE neuroimaging studies suggest the existence of sex differences in the spatial distribution of brain alterations with men more often showing frontal lobe dysfunction ipsilateral to the seizure onset zone and women exhibiting dysfunction in the contralateral temporal lobe [179, 180].

Differences in prognosis have been found for specific epileptic syndromes.

Among IGE, a systematic review found later response to anti-seizure medications (ASMs) [181], greater risk of relapse after ASMs withdrawal [182] and overall worse seizure control [183] in women with Juvenile Myoclonic Epilepsy (JME) [184]. Moreover, sex differences in prescribing patterns such as reduced use of valproic acid (VPA) or its use at lower dosages in women could negatively influence the outcome [184, 185]. The worse outcome in women with IGE was confirmed, irrespective of VPA use, by a prospective observational study [186].

The excess in mortality in epilepsy is higher for men, as demonstrated in different studies, as well as the risk of suicide which is increased almost sixfold in men but not in women [187]. However, the risk of sudden unexpected death in epilepsy (SUDEP) is not different between men and women [188].

#### Treatment

Two recent systematic reviews investigated sex differences in adverse effects of ASMs, suggesting a higher frequency of general, cutaneous and metabolic adverse effects of ASMs in female gender both in pediatric and adult patients with epilepsy. The effect of sex and gender on the response to ASMs has been investigated by a recent systematic review showing that most studies do not identify statistically significant differences. Among those which found a different response to ASMs, a greater number of studies reported higher rates of seizure reduction or seizure freedom in males using different ASMs [189–191].

#### Pregnancy and breastfeeding

Epilepsy presents unique challenges during pregnancy, requiring comprehensive care to protect both maternal and fetal health. Seizure management remains the principle of care, as poorly controlled epilepsy can lead to maternal injuries, fetal hypoxia, and even fetal or maternal mortality in extreme cases [191, 192]. The physiological changes during pregnancy, including increased plasma volume, heightened renal clearance, and altered protein binding, significantly impact the pharmacokinetics of ASMs. These changes demand careful monitoring and dose adjustments to maintain therapeutic levels of medication [193]. Notably, lamotrigine and levetiracetam exhibit increased clearance during pregnancy, particularly in the first trimester, necessitating dosage revisions to prevent breakthrough seizures [194, 195]. Conversely, unbound levels of drugs such as valproate, which are pharmacologically active, require close monitoring due to potential teratogenic risks [196].

The use of certain ASMs during pregnancy is associated with varying degrees of teratogenic risk. Valproate, for example, carries one of the highest risks, with major congenital anomalies (MCAs) occurring in up to 25% of exposed pregnancies at higher doses (> 1450 mg/day) [197]. Beyond MCAs, valproate exposure is linked to neurodevelopmental disorders in offspring, such as autism spectrum disorder (ASD), intellectual disability, and attention deficit hyperactivity disorder (ADHD) [198]. This has led to stringent restrictions on its use in people of childbearing potential [199]. Other ASMs, like carbamazepine, phenobarbital, and phenytoin, also carry moderate teratogenic risks, though less severe than valproate [200, 201]. Meanwhile, medications like lamotrigine and levetiracetam are considered relatively safer, with lower risks of MCAs and no consistent evidence of adverse neurodevelopmental outcomes [202, 203]. However, emerging data on topiramate suggest it may increase risks for both MCAs and adverse neurodevelopmental outcomes, further highlighting the need for careful ASM selection [204].

The absence of robust data for newer ASMs or polytherapy regimens underscores a significant gap in current knowledge. For instance, oxcarbazepine and zonisamide show some association with fetal growth restriction, yet long-term impacts remain unclear [203, 205]. This emphasizes the importance of patient-specific counseling and risk-benefit analyses, guiding decisions on ASM use in pregnancy.

Folic acid plays a critical role in reducing the risk of neural tube defects (NTDs) in the general population and is especially vital for people with epilepsy due to the potential adverse effects of ASMs on folate metabolism [206, 207]. Many ASMs, particularly enzyme-inducing drugs like phenytoin and carbamazepine, can deplete folate levels, increasing the risk of fetal malformations [202]. While folic acid supplementation is universally recommended for people planning pregnancy, the optimal dosage for those on ASMs remains a subject of debate [199, 208], ranging from 0.4 mg to 5 mg daily [209]. The World Health Organization (WHO) recommends for healthy women a baseline supplementation of 0.4 mg daily, starting at least three months before conception and continuing through the first trimester to prevent NTDs [210]. The practice guidelines of the American Academy of Neurology, published in 2025 [211], state that clinicians are required to prescribe at least 0.4 mg of folic acid supplementation daily, both preconceptionally and during pregnancy, to any person with epilepsy on ASM treatment, in order to potentially improve neurodevelopmental outcomes, including autism spectrum disorder and overall IQ in the offspring. However, some studies suggest that higher doses may be necessary for those on specific ASMs or with a history of NTDs in prior pregnancies [212].

Emerging research highlights a potential U-shaped relationship between folic acid levels and outcomes, where both deficiency and excessive intake might carry risks [213, 214]. High doses, for instance, have been linked to poorer cognitive outcomes in children and a potential increase in maternal and fetal cancer risks, though these findings require further investigation [215]. Tailored supplementation strategies, accounting for individual risk factors such as ASM type, adherence, and baseline folate levels, may optimize outcomes. Routine measurement of serum folate and vitamin B12 levels could further guide supplementation, ensuring adequate yet safe levels are maintained [216, 217].

A coordinated, multidisciplinary approach is essential during labor and delivery to mitigate risks associated with epilepsy [209]. Continuous ASM administration and fetal monitoring are critical during labor to prevent seizure exacerbation and ensure fetal well-being [199]. The mode of delivery—vaginal or cesarean—should be determined based on obstetric and neurological considerations, with cesarean sections reserved for individuals with poorly controlled seizures or other complications [218]. Stress reduction techniques, including epidural anesthesia, can help minimize seizure risks during labor [219].

Postpartum, the immediate adjustment of ASM dosages to pre-pregnancy levels is necessary to address the pharmacokinetic reversals after delivery [193]. Sleep deprivation, common in new parents, is a significant seizure trigger; thus, strategies to ensure adequate rest and support are crucial [220]. Concentrations of ASMs are detectable in breastmilk and, consequently, in breastfeeding infants [192, 199, 221]. For most ASMs, other than ethosuximide, phenobarbital, and zonisamide, very low to low concentrations in breastfed infants are reported (10–30% of maternal serum levels) [199]. Acute side effects are rare in breastfed infants, and there is evidence that breastfeeding does not adversely affect neurodevelopment up to 6 years of age [192, 202, 222]. For these reasons, breastfeeding should be encouraged in people with epilepsy [199, 209].

A summary of available evidence and knowledge gaps are presented in Table 4.

**Table 4:** Main sex/gender differences in epilepsy and knowledge gaps

Topic	Summary of available evidence	Knowledge gaps
Epidemiology	Epilepsy is slightly more frequent in men due to higher exposure to risk factors, while Idiopathic Generalized Epilepsies (IGE) are more common in women, with a female-to-male ratio of ~ 1.5.	Lack of sex-disaggregated data on incidence, prevalence and subtype of distribution, especially in underrepresented populations and across the lifespan.
Pathophysiology	Several biological factors—including hormones, enzyme activity, and neuronal circuitry—affect seizure susceptibility. In women, hormonal fluctuations (e.g., during menstrual cycle, pregnancy, menopause) can alter seizure threshold.	Limited understanding of molecular sex-specific mechanism underlying seizure susceptibility and drug response.
Clinical presentation	In temporal lobe epilepsy (TLE), focal aware seizures are more frequent in women, while bilateral evolution is more common in men. Men with generalized epilepsy show more atonic seizures, whereas women report more autonomic, visual, and psychic symptoms in focal epilepsy.	Further investigations into seizure propensity, differential clinical presentation as well as gender-based impact on quality of life and comorbidities are warranted.
Treatment	In IGE, women seem to have delayed response to ASMs, higher relapse rates after withdrawal, and poorer seizure control. Systematic reviews show women—both pediatric and adult—experience more adverse effects. No significant differences are found in drug response but males seem to show a better outcome in few studies. Pregnancy management is complex due to ASM pharmacokinetics and teratogenic risks.	Further investigations are needed to personalize therapeutic approaches.
Pregnancy & Breastfeeding	Management requires careful ASM monitoring and dose adjustments due to pharmacokinetic changes and teratogenic risks. Valproate carries the highest teratogenic risk; lamotrigine/levetiracetam are relatively safer. Folic acid supplementation is vital for preventing neural tube defects. Breastfeeding is generally encouraged as ASM levels in breastmilk are low, with rare infant adverse effects.	Lack of robust data on newer ASMs/polytherapy safety and long-term in utero exposure effects. Further research is also needed to optimize folic acid supplementation strategies.

#### Recommendations and future directions

- Provide an individualized approach to balance seizure control with the minimization of fetal risks during pregnancy.
- Improve monitoring for ASMs and expand research into the long-term effects of in utero ASM exposure, that are critical for advancing care [192, 222].
- Improve access to safer ASMs and comprehensive reproductive counseling, particularly in resource-limited settings [223].
- Promote research on the safety profiles of newer ASMs, both as monotherapy and polytherapy regimens, during pregnancy. Understanding the potential risks associated with these medications, such as the emerging concerns regarding topiramate, is crucial for optimizing treatment strategies.
- Promote research on optimizing folic acid supplementation strategies, considering individual risk factors and the potential for both deficiency and excessive intake to impact maternal and fetal health. Longitudinal studies examining the neurodevelopmental outcomes of children exposed to different folic acid supplementation levels in utero are necessary to clarify current recommendations.
- Promote further investigation into sex differences in epilepsy, including prevalence, seizure propensity, response to therapy, and susceptibility to adverse effects of ASMs, is warranted to personalize treatment approaches.

### Headache disorders

#### Epidemiology

Primary headache disorders have a clear sex difference in their prevalence, although with a varying direction. While migraine and tension-type headache are common diseases and by far more prevalent in women, cluster headache is a less common disorder, which is more prevalent in men [224, 225]. Of note, the women prevalence of migraine is dependent on the phases of life, being similar in both sexes until adolescence, prevailing in women during their fertile age, with a peak in the second and third decades, followed by a reduction in both sexes after the fifth decade [226, 227].

#### Risk factors and pathophysiology

Primary headache disorders are influenced by both genetics and sex hormones. The aggregation of headache disorders in families is well-known; familiar aggregation of headache disorders has a dominant behavior, as those disorders tend to present themselves in all generations of a family. The different sex prevalence of headache disorders like migraine and cluster headache is likely due to different penetrance rather than to different mechanisms of gene transmission to sexes [226].

Sex hormones play an important role in the different sex prevalence of some headache disorders. In this respect, the most studied headache disorder is migraine. The brain has widespread receptors for female sex hormones, that can modulate the activity of many areas implied in the pathogenesis of migraine, including the trigeminovascular system and limbic structures such as the amygdala [228]. The periodic falls in estrogen levels that accompany the pre-menstrual period of the ovarian cycle are linked to an increased susceptibility to migraine attacks, as a possible consequence of a decreased inhibition of pain perception [229]. This mechanism might also explain the increased susceptibility to migraine attacks in women treated with combined oral contraceptives and especially those with high estrogen content [230]. While female sex hormones, and especially estrogens, are linked to an increased susceptibility to migraine, male hormones are associated to a decreased susceptibility to migraine attacks. A small case series of women with migraine and breast cancer treated with male sex hormones because of their cancer showed that all women became migraine-free after receiving hormonal treatment [231]. Some migraine-specific pathophysiological mechanisms such as calcitonin gene-related peptide (CGRP) expression are also influenced by female sex hormones; higher levels of CGRP are found in female compared with male animals in preclinical models [232]. Migraine is the headache disorder in which hormonal influences were studied the most; however, sex hormones might also influence differences between men and women in other headache disorders.

Besides genes and sexual hormones, other factors are believed to influence sex differences in the presentation of primary headache disorders. Again, those factors have been extensively studied in migraine, while their investigation is lacking for other headache disorders. Migraine can be considered a bio-psycho-social disorder in which individual susceptibility sums up with psychological factors and sociocultural context [233]. Data from the large CaMEO study showed that the burden of migraine is overall higher in women than in men and that the prevalence of some comorbidities such as asthma and anxiety is also higher in women, while hypertension and obesity are more prevalent in men than in women [234]. On the other hand, psychosocial factors can also contribute to sex differences, such as stigma towards migraine as a “female illness”, which might lead to poorer pain reporting and healthcare seeking in men compared with women [234].

#### Clinical features

Increasing evidence suggests that some primary headache disorders manifest with a sex-related phenotype. In the case of migraine, the typical accompanying symptoms tend to be more expressed in women than in men and the burden of disease is higher in women, while men tend to receive a more delayed diagnosis of migraine and are prescribed less treatments than women [226, 234, 235]. In the case of cluster headache as well, women are generally more burdened by symptoms than men [236]; moreover, there is a higher prevalence of symptoms such as nausea and osmophobia in women than in men, configuring a “migraine-like” phenotype in women [237]. As for other headache disorders, there are no studies on sex differences in their clinical presentation.

#### Treatment

Very few studies have investigated sex differences in the pharmacokinetics or efficacy of headache treatments, and they all focused on migraine. A systematic review on sex-related response to triptans found no substantial sex differences in the efficacy of the drugs except for a lower proportion of headache recurrence in men compared with women; however, adverse events associated with triptan use were more frequent in women compared with men [238]. Notably, the exposure to triptans was lower in men compared with women, which might have influenced the results [238]. A multicenter study on onabotulinumtoxinA treatment showed no sex differences in effectiveness or safety of the drug, while men showed a higher rate of treatment discontinuation compared with women [239]. Referring to treatments targeting the calcitonin gene-related peptide (CGRP), observational studies showed no difference between men and women related to monoclonal antibodies [239], while the efficacy of gepants – oral antagonists of the CGRP receptor – is lower in men than in women according to clinical data [240]. This sex-related difference is consistent with animal studies showing higher peripheral CGRP expression in females, which may explain why CGRP blockade is more effective in women [232].

#### Pregnancy and breastfeeding

Addressing the relationship between primary headache disorders and pregnancy, pregnancy planning, and breastfeeding is relevant from a public health perspective, particularly for migraine, because it is a highly disabling condition with the highest prevalence in women during their fertile period of life [225]. While migraine generally improves during pregnancy, this is not true for all women, especially those suffering from migraine with aura [241, 242]. In addition, a residual, clinically relevant burden may persist also in women who report a pregnancy-related improvement, so that acute or even preventive treatment may be required.

As regards breastfeeding, it may be protective in a large portion of women [243], probably because of high prolactin levels, with the consequent amenorrhea. However, migraine women exclusively breastfeed their infants for a shorter duration compared to women without migraine, possibly as a consequence of the perceived poor safety of breastfeeding when having migraine attacks [244].

Unfortunately, none of the drugs that proved effective in the acute or preventive treatment of migraine can be considered entirely safe during pregnancy, although acetaminophen (paracetamol) for the acute treatment and propranolol or low-dose amitriptyline for the preventive treatment can be used, after a careful risk/benefit evaluation [242]. Locally acting injectables such as local anesthetics [245] or onabotulinumtoxinA [246] are also assumed as safe given their lack of systemic effects, as well as non-invasive neuromodulation.

A summary of available evidence and knowledge gaps are presented in Table 5.

**Table 5:** Main sex/gender differences in primary headache disorders and knowledge gaps

Topic	Summary of available evidence	Knowledge gaps
Epidemiology	Migraine and tension-type headache have their highest prevalence in young women.	Few epidemiological data on rare headache disorders.
Pathophysiology	The presence of sex hormone receptors in the brain influences the women’s susceptibility to migraine.	Exact mechanisms underlying the sex hormone-related susceptibility to migraine; sex-specific targets for treatment.
Clinical presentation	Migraine phenotype is characterized by a higher number of associated symptoms in women than in men. Cluster headache in women share more similarities with migraine.	Data on sex-related differences in tension-type headache; prospective data on sex- and gender-related outcome of disease; sex-related predictors of disease progression.
Treatment	Women might respond better than men to migraine medication such as triptans and gepants, given their sex-specific expression of molecules implied in migraine pain.	Biomarkers of response to treatment.

## Recommendations and future directions

- Migraine may be a progressive disorder characterized by an increasing frequency and severity of attacks, which can lead to the overuse of acute medications and a progressive reduction in their efficacy. This phenomenon is mostly observed in women. Therefore, clinicians should identify any worsening of the disease early on, implementing an effective preventive treatment combined with educating patients about the risks of increased use of acute medications.
- While hormonal therapy can potentially worsen migraine, it can also help reduce the burden of the disease in some cases. This is particularly true for menstrual-related migraine with long-lasting, treatment-resistant attacks that recur during menstruation. In this case, consulting a gynecologist who specializes in headaches can be very helpful.
- Migraine usually improves or remits during pregnancy. However, for women whose migraines do not improve or even worsen during pregnancy, a careful approach should be adopted, whereby the risks and benefits of taking antimigraine treatments are explained and discussed with the patient.
- Tension-type headache can be aggravated by stress and psychological comorbidities; therefore, in patients with chronic tension-type headaches (defined as having at least 15 headache days per month for at least three months), a non-pharmacological approach based on behavioral therapy can be helpful.
- Future research should cover multiple areas. Firstly, there is a clear need for studies on sex differences in primary headache disorders, as well as on their management during pregnancy and breastfeeding. Secondly, innovation in the detection of multimodal biomarkers, supported by large data lakes and artificial intelligence, should help us to understand more precisely how the multiple structures and systems involved in the pathophysiology of primary headaches interact with each other, and how sexual hormones mediate such processes. A better understanding of the interaction between genetic and epigenetic factors will hopefully lead to more targeted treatments. Substantial contributions to disentangling sex-related determinants within the complex processes underlying migraine pathophysiology will likely be provided by available, reliable animal models of migraine.
- In terms of public health, awareness campaigns and dedicated healthcare policies are needed to promote correct and early diagnosis, especially in the case of cluster headache, and to improve access to treatment and discourage self-medication and acute medication overuse.

### Multiple sclerosis

#### Epidemiology

Multiple sclerosis (MS) is a chronic inflammatory disease of the central nervous system characterized by multifocal demyelination and neurodegeneration [247]. Originally reported in the 1940 s to have a 1:1 female-to-male (F: M) ratio, subsequent epidemiological studies have revealed a progressive skew toward a ratio approaching or even exceeding 3:1 [248]. This shift is driven by an increased MS rate in women, with a stable rate in men [249].

While a combination of genetic, epigenetic, and environmental factors may contribute to these dynamics, the rapid rise in incidence over the past century argues against a predominantly genetic origin (genetic drift), suggesting a crucial interaction between sex and environmental exposures [250].

#### Risk factors and pathophysiology

A notable sex-specific finding in MS research is the identification of a genome-wide significant susceptibility locus on the X chromosome. The variant rs2807267, which confers an odds ratio of 1.07 (*p* = 6.86 × 10^−^⁹), is located within a T cell–specific enhancer peak [251]. No significant associations have been observed on the Y chromosome, underscoring the potential contribution of X-linked genetic factors to the marked women preponderance observed in MS.

Beyond genome-wide association studies (GWAS) evidence, inherent disparities in sex chromosome composition give rise to critical differences: (1) variations in X gene dosage, (2) differential maternal versus paternal X imprinting, and (3) the presence or absence of Y-linked genes [252, 253]. Experimental models, such as the Four Core Genotype mice, underscore the impact of these chromosomal factors—illustrated by the involvement of genes like Kdm6a [254] —on the proinflammatory profile seen in MS, highlighting a critical role of genetic factors in sex-specific disease mechanisms.

Beyond improved diagnostic accuracy, the rapid rise in MS incidence over the last century points to a critical role for environmental factors underlying the heightened incidence in women [255].

Lifestyle changes over recent decades have played a significant role [256]. Women with MS (WwMS) have experienced a shift toward later age at first pregnancy, lower childbirth rates, and higher levels of employment and smoking [255, 256].

Modern shifts away from outdoor lifestyles may be reducing serum vitamin D levels, with experimental models suggesting that vitamin D may uniquely protect WwMS from autoimmune demyelination [257]. Low sunlight exposure—and potential sex-specific effects of ultraviolet radiation—has likewise been associated with heightened MS risk [257]. Finally, sex differences in gut microbiome regulation—which drives hormone-dependent modulation of autoimmunity—have been reported [258] and may also contribute to the observed imbalanced ratio.

Although the pronounced women predominance in MS is established, it is noteworthy that this difference arises only after puberty—prior to which incidence is nearly equal. This timing implicates hormonal changes during puberty as pivotal in influencing MS risk [259–261]. An earlier onset of menarche correlates with an increased risk and a younger onset of MS [262, 263], around two years post-menarche [264]. In addition, women who have not experienced pregnancy appear to face a higher risk of MS compared to those with multiple pregnancies [265]—a contrast that may be attributed to the biphasic immunomodulatory effects of estrogens, which can stimulate immune activity at low levels and suppress it at high levels typical of pregnancy [266, 267].

Hormonal influences continue beyond the reproductive years, with menopause representing another key transition [268]. Although women predominance and relapse rate decrease after menopause [269], some studies suggest an acceleration of disability progression in postmenopausal women [270–272]. These effects may be mediated by declining estrogen and progesterone levels, which contribute to immunosenescence, reproductive senescence, and neurodegeneration - including grey matter loss and increasing disability [268]. However, it remains unclear whether menopause directly causes these changes or merely parallels age-related decline [268].

#### Clinical and radiological features

While women are at higher risk of developing MS and experiencing relapses [273], men tend to exhibit a more aggressive disease course [274, 275], a progressive onset [276], poorer recovery following initial relapses, more rapid disability accrual, with a steeper increase of the Expanded Disability Status Scale score [277]; they also have a higher likelihood of transitioning to secondary progressive MS [278], along with greater brain volume loss and more severe cognitive impairment [279].

In the context of MS, the existence of consistent sex-based MRI differences remains controversial. A 2009 literature review reported no significant differences in T2 or T1 lesion burden, brain atrophy, magnetization transfer ratio, or diffusion tensor imaging metrics between the sexes [280]. Nonetheless, some studies suggest that men may exhibit fewer contrast-enhancing lesions but demonstrate greater gray matter volume loss, increased T1 hypointense lesion load, and enhanced spinal cord axon loss [281]. Additionally, men have been reported to experience greater retinal nerve fiber layer thinning following optic neuritis [282]. One study indicated that while gray matter and central atrophy were more advanced in men, white matter atrophy was more pronounced in women [283]. A more recent investigation also documented greater regional thalamic and cortical gray matter atrophy in men with MS [284].

This domain clearly warrants large-scale studies, potentially incorporating machine learning techniques, to elucidate the underlying mechanisms and further characterize these sex-specific neuroimaging findings.

#### Treatment

Beyond differences in disease risk and course, significant sex-related differences have also recently emerged in treatment strategies. A large real-world analysis of 4,224 MS patients from the Austrian Multiple Sclerosis Treatment Registry found that WwMS experienced longer delays before initiating disease-modifying therapies (DMTs) and showed slower treatment escalation in response to relapse activity—with an increase in annual relapse rate resulting in about a fourfold escalation risk in women compared to an eightfold increase in men [285]. Additionally, younger women were more likely to discontinue moderate-efficacy DMT, and overall, women had a higher propensity to stop high-efficacy treatments, largely due to family planning considerations [285]. These findings suggest that treatment decisions are not solely driven by clinical factors such as relapse rate or disability progression but are also influenced by sex-specific issues.

#### Pregnancy and breastfeeding

Pregnancy confers protection against MS relapses by inducing hormonal shifts—mediated by estrogen, progesterone, and hCG—that promote a tolerogenic immune environment characterized by a mid-gestation shift from Th1 to Th2 responses, altered T cell clonal composition [286–288], and variable modulation of regulatory T cells and natural killer cell populations [289–293]. However, pre-pregnancy immunity is restored postpartum, often correlating with the sensible risk of disease reactivation [294].

Thus, family planning in MS requires a comprehensive, individualized strategy that begins at diagnosis and extends through childbearing age. WwMS should undergo preconception counseling, based on a detailed clinical and MRI evaluation, lifestyle and preferences, to devise the best personalized treatment strategy aimed at maintaining the highest quality of life.

Contraceptive counseling is essential in MS care, especially before initiating or modifying DMTs. Tailored discussions about long-acting reversible methods—such as intrauterine devices and subdermal implants—should occur at diagnosis or prior to any therapy change. Combined hormonal contraceptives are best avoided in patients with elevated venous thromboembolism risk or significant immobility, and depot medroxyprogesterone acetate may be contraindicated in those with low bone density. Male contraception should also be addressed, given potential DMT-mediated fetal risks. Clinicians must coordinate DMT washout intervals with reliable contraceptive cover and remain vigilant for drug–hormone interactions (e.g., S1P modulators reducing oral contraceptive efficacy). Engaging patients in shared decision-making optimizes both neurological and reproductive outcomes [295].

In managing DMT choice, clinicians must weigh the teratogenic risks—particularly with S1P receptor modulators (fingolimod, ozanimod, ponesimod)—and the risk of disease reactivation or rebound associated with these agents and natalizumab—against the safety profiles of injectables (interferon β, glatiramer acetate), which may be continued, in WwMS with milder disease activity, throughout pregnancy. Natalizumab carries a known reactivation risk if discontinued, yet—because placental FcRn-mediated transfer is negligible during the first trimester—it is often maintained throughout pregnancy to preserve disease control, with careful monitoring for hematologic changes in the newborn [296]. By contrast, anti-CD20 antibodies (e.g., ocrelizumab) have traditionally required a 6–12-month washout before conception, but emerging registry data support shortening this window to conception, without increasing rates of spontaneous abortion or major congenital anomalies, since placental transfer of IgG1 only becomes significant after ~ 20 weeks’ gestation [297]. Cladribine, with its long-lasting efficacy, allows pregnancy planning from 6 months after the final dose of the 2-year course, without the need for bridging therapies or concerns of rebound activity [298]. Overall, for women with active disease, transitioning to highly effective, low-rebound therapies (e.g., depleting antibodies) may offer superior disease control while minimizing fetal exposure whereas those with milder disease might safely maintain injectable therapies.

Postpartum, careful planning is essential; breastfeeding offers both immunologic protection for the infant and a modulatory effect on postpartum MS activity, and recent data support its safety alongside selected DMTs [299]. Meta-analyses show that exclusive breastfeeding reduces relapse risk in the first six months postpartum and is associated with fewer new MRI lesions [300, 301]. In deciding when to resume therapy, molecular weight and oral bioavailability govern breastmilk transfer: large, poorly absorbed injectables (interferon-β, glatiramer acetate; RID < 1%) and IgG-based monoclonals (natalizumab, rituximab, ocrelizumab; RID < 1%) are compatible with continued breastfeeding [296]. By contrast, small oral agents (e.g. cladribine, dimethyl fumarate) exhibit higher transfer or unknown profiles and are generally avoided until weaning [288]. Shared decision-making—balancing maternal relapse risk, infant exposure, and the wide-ranging benefits of breastfeeding—should guide individualized postpartum DMT plans, ideally within a multidisciplinary mother–infant care framework.

Assisted reproductive technologies (ART) should also be an integral part of pregnancy planning in MS. WwMS, who often delay conception until disease and treatment stability are achieved, are more likely than their peers to require assisted reproductive technologies (ART) such as intrauterine insemination, IVF/ICSI, and oocyte cryopreservation [302]. ART procedures do not appear to increase relapse risk when appropriate ovarian-stimulation protocols (e.g. GnRH-antagonist–based) are used [303], and live-birth rates are comparable to those in women without MS [304]. Early discussion of fertility preservation (such as oocyte vitrification before age-related decline) and close collaboration between neurology and reproductive-medicine specialists are therefore essential to optimize both neurological stability and reproductive outcomes [302, 305].

A summary of available evidence and knowledge gaps are presented in Table 6.

**Table 6:** Main sex/gender differences in multiple sclerosis and knowledge gaps

Topic	Summary of available evidence	Knowledge gaps
Epidemiology	MS initially had a reported 1:1 female-to-male ratio in the 1940 s but has since shifted toward 3:1 (or higher), driven by increasing incidence in women while rates of men remain stable.	Detailed epidemiology in non-Caucasian and low-prevalence populations.
Genetic & epigenetic factors	A genome-wide significant susceptibility locus on the X chromosome (rs2807267, OR 1.07, *p* = 6.86 × 10⁻⁹) highlights X-linked contributions. Four Core Genotype mouse models (e.g., Kdm6a) demonstrate how sex chromosome complement influences pro-inflammatory profiles in MS.	Molecular and functional mechanisms of X-linked variants across MS subtypes; roles of maternal versus paternal X imprinting in female susceptibility.
Environmental factors	Lifestyle shifts - later first pregnancies, reduced UV exposure (and thus lower vitamin D) - plus sex-specific gut microbiome changes have been implicated in the rising women incidence of MS.	The role of widespread hormonal contraceptive use, especially in primary progressive MS. Specific interactions between environmental exposures (e.g., vitamin D × sex hormones); specific interactions between environmental exposures and genetic regulation (e.g. obesity x gene expression).
Hormonal influences	The post-pubertal rise in women’s MS incidence and earlier menarche’s correlation with earlier MS onset implicate estrogen and progesterone. Menopause is associated with reduced women’s predominance but possible acceleration of disability progression, likely tied to declining sex steroids, which contribute to immunosenescence, reproductive senescence, and neurodegeneration.	Exact mechanisms of pre-pubertal protection and post-menopausal decline; direct causal links between menopause and disability progression versus age-related effects.
Clinical & radiological presentation	Women have higher relapse rates throughout MS, but men tend to show more aggressive progression: poorer relapse recovery, faster EDSS worsening, earlier transition to SPMS, greater brain volume loss, and more severe cognitive decline. Neuroimaging findings are mixed, though some studies suggest men exhibit greater gray-matter atrophy, higher T1 lesion load, and more spinal cord damage, while women may show more white-matter atrophy.	Large-scale, sex-stratified MRI studies using advanced methods (e.g., machine learning) to clarify sex differences in lesion distribution, atrophy patterns, and microstructural damage.
Treatment & management	Real-world data show women face longer delays to DMT initiation, slower treatment escalation following relapses, and higher discontinuation rates—often due to family planning. Contraceptive counseling in MS should be individualized, initiated early, and coordinated with DMT plans to ensure safety, efficacy, and alignment with reproductive goals. Pregnancy planning in MS demands weighing the high rebound risk of natalizumab and fingolimod against the more stable profiles of anti‑CD20s and cladribine. Breastfeeding and ART considerations further influence therapy choices in women.	Focused trials on optimal DMT sequencing by sex; biology-driven guidelines for pregnancy, postpartum, and breastfeeding; sex-specific evaluation of ART protocols and long-term reproductive outcomes.

#### Recommendations and future directions

- Consider sex and gender as key determinants of interindividual differences in clinical presentation, neuropathology, treatment response and the risk of disability accrual in MS.
- Consider that the incidence and prevalence of MS is higher in women than in men, but disability accrual is generally worse in men.
- Consider sex-specific risk factors including pregnancy, puerperium and menopause that warrant peculiar attention and consideration.
- Consider post-hoc analysis of previously published clinical trials or meta-analysis of data already available to increase knowledge of sex differences in efficacy and safety of DMTs.
- Consider a tailored, sex-informed approach to deliver individualized care to persons with MS (PwMS). In particular:
  - Provide an individualized approach to balance disease control with the minimization of fetal and maternal risks during pregnancy.
  - Improve knowledge about DMT exposure during pregnancy and breastfeeding and long-term effects of fetal and neonatal DMT exposure, which are critical for advancing care.
  - Improve referral and access to high efficacy therapies for WwMS.
- Sustain public health interventions targeting modifiable risk factors, to decrease disease severity and progression.
- Promote patient engagement in all research and healthcare processes involving PwMS.
- Promote inclusion of sex in clinical trial design, ensuring a proper stratification of men and women into experimental groups and pre-specified ad-hoc analyses of sex subgroups for both efficacy and safety of the drug under development.
- Promote development of comprehensive and rigorous recommendations to ensure that sex and gender are appropriately investigated and reported in clinical trials, as well as in the diagnosis and treatment of MS. Such efforts could pave the way for more inclusive and targeted intervention strategies, and paradigm shifting policies that may improve our ability to timely prevent, diagnose and treat MS.

### Overview of evidence in sex and gender minorities

Differences in the epidemiology, pathophysiology, clinical course, and management of diseases among gender minorities remain an area of ongoing research, and the evidence so far available is generally poor.

#### Alzheimer’s disease

There is an expanding field of research on cognitive decline in sexual and gender minorities (SGM), but the knowledge gap is still far from being filled. Evidence from studies in non-heterosexual populations demonstrates how individuals who self-identify as belonging to a sexual minority suffer from “minority stress”, possibly worsening their cognitive trajectories [291]. Moreover, SGM individuals face a higher burden of mental health issues, cardiovascular risk factors, and social isolation, which may interact with biological vulnerability to accelerate cognitive decline [292]. Nonetheless, the scarcity of clinical and biomarker-based studies prevents clear conclusions about disease mechanisms or differential dementia risk, and calls for an integration among social and clinical neuroscience perspectives.

#### Cerebrovascular disease

A retrospective case–control study of 26 SGM patients and other small case series reported a similar distribution of stroke subtypes compared with cisgender controls, but a younger age at onset and a distinct risk factor profile, including higher rates of HIV infection, syphilis, and hepatitis C, although these conditions were more frequently screened for in this population [293, 306].

Long-term exposure to gender-affirming hormone therapy (GAHT) in transgender individuals represents another relevant risk factor, as it has been associated with an increased likelihood of cerebrovascular events. Accordingly, the continuation of such therapy should be carefully reassessed following an acute stroke [307]. A meta-analysis focusing on male-to-female (MTF) transgender individuals reported an overall incidence of cerebrovascular events of approximately 2% during estrogen therapy [308].

Complementing these smaller studies, a recent population-based analysis of the 2020–2022 Behavioral Risk Factor Surveillance System identified 189 transgender stroke survivors and demonstrated higher odds of stroke compared with cisgender individuals (aOR = 1.69). These individuals also exhibited a greater prevalence of modifiable lifestyle-related risk factors, including e-cigarette use, tobacco smoking, alcohol consumption, and binge drinking, highlighting the potential impact of behavioral factors on stroke risk [309].

Despite these findings, current evidence is limited by small sample sizes, heterogeneous study designs, and inadequate adjustment for confounders, while data on acute stroke management and secondary prevention in transgender and nonbinary populations remain scarce. Larger prospective studies are needed to better define stroke risk and guide preventive and therapeutic strategies.

#### Parkinson’s disease

Concerning PD patients, data on SGM populations remain limited. A survey conducted in a specialized movement disorders center reported a prevalence of SGM individuals up to 4.3% among patients with various movement disorders [310]. A similar prevalence was found in an online survey of people with PD (the Fox Insight cohort), showing that SGM participants experienced higher levels of discrimination when using healthcare services compared to non-SGM peers [311]. More recently, analyses from the same cohort revealed that SGM individuals with PD face specific barriers to research participation and may be at higher risk of cognitive functional impairment, particularly among those assigned male at birth [312, 313]. These findings underscore the need to better characterize and address the specific clinical and psychosocial needs of SGM populations within PD care and research.

#### Epilepsy

Recent studies have begun to address the health disparities affecting SGM individuals with epilepsy, a topic historically underrepresented in the literature [314]. A national study based on the 2022 U.S. National Health Interview Survey found that SGM adults—defined as individuals identifying as transgender, gender-diverse, or reporting non-heterosexual orientations—were twice as likely to report active epilepsy compared to non-SGM adults (adjusted OR 2.14; 95% CI 1.35–3.37) [315]. This disparity persisted even after adjusting for sociodemographic variables and was moderately attenuated when controlling for depression (adjusted OR 1.67; 95% CI 1.00–2.60).

SGM individuals with epilepsy face an elevated burden which is multifactorial and include double stigma related to both epilepsy and gender identity, barriers to care, and chronic stress linked to discrimination [316].

In transgender patients, GAHT may interact with ASMs, particularly enzyme-inducing drugs, necessitating careful monitoring of serum levels of agents like lamotrigine and valproic acid [317, 318]. Moreover, transgender individuals with epilepsy face increased risks of comorbidities such as depression, suicidality, substance abuse, and low bone mineral density, which may be exacerbated by both GAHT and ASMs [318, 319].

Despite these concerns, clinical research remains scarce, and no interventional studies have yet evaluated epilepsy outcomes in lesbian, gay, bisexual, transgender, queer or questioning, plus other identities (LGBTQ+) populations [320]. This gap underscores the urgent need for inclusive research and clinician training to improve care for these vulnerable groups [321].

#### Headache disorders

The prevalence of headache disorders among individuals undergoing GAHT varies from 6.8% [322] to 63.7% [323]. A cross-sectional study reported migraine prevalence of 36.4% and tension-type headache prevalence of 40.9% among female-to-male individuals experiencing gender dysphoria [324]. Interestingly, transgender women treated with estrogens showed a headache prevalence similar to that of cisgender women [325], which confirms the role of estrogens in headache disorders such as migraine. The use of male sex hormones for GAHT has been associated to an increase in migraine prevalence [326]. However, a case-control study showed that estrogen-based feminizing hormone treatments tend to worsen headache, while testosterone-based masculinizing treatments may lead to headache improvement [322, 323].

Notably, transgender individuals face a high burden of psychiatric comorbidities including anxiety, depression, and substance use disorder, together with stigma, discrimination, and barriers to care; all those factors can exacerbate migraine [327].

Regarding treatment, care should be taken when using anticonvulsants to prevent migraines in individuals undergoing masculinizing hormone treatment, as these drugs can affect testosterone levels [322].

#### Multiple sclerosis

Multiple sclerosis at the intersection with LGBTQ + health requires attention to both biological considerations and healthcare equity [328, 329]. Biologically, sex hormones influence multiple sclerosis susceptibility and disease activity; pregnancy-level estrogens are associated with reduced relapse risk, selected estrogen formulations have shown neuroprotective signals, and low testosterone in men with multiple sclerosis relates to worse outcomes—all of which supports a plausible role for exogenous hormones used in gender-affirming care [329]. Emerging human data indicate that after feminizing transition, diagnoses of multiple sclerosis have been observed more frequently than expected, whereas masculinizing transition does not show a comparable increase, suggesting that lowering testosterone and adding estrogen could, in a subset of susceptible individuals, tilt toward greater risk [330]. Among people already living with multiple sclerosis, small retrospective series report new relapses or MRI activity in some individuals within months of starting GAHT, with progression noted in a subset of transgender women; in contrast, cohorts composed primarily of individuals assigned female at birth receiving testosterone have shown mild disability overall and no signal of increased relapses, progression, or radiologic activity [331, 332]. These findings are preliminary and limited by sample size and design, but they support a practical, patient-centered approach: do not discourage necessary GAHT; obtain a thorough gender and hormone history; coordinate with endocrinology; and consider closer clinical and MRI follow-up around major hormonal changes [328, 331]. In parallel, LGBTQ + people with MS experience measurable disparities. They report less comfort discussing sexual health, slightly lower satisfaction with clinicians, more frequent switching of providers when clinic climates feel unfriendly, and fewer mental-health visits [328, 333–335]. Transgender and bisexual individuals carry particularly high burdens of anxiety, depression, suicidality, and substance use, which can compound MS fatigue, pain, and cognitive symptoms if unrecognized [328]. Delays in disclosure or care—driven by stigma, misgendering, or heteronormative assumptions—risk later diagnosis or preventable relapses [328]. Addressing these gaps does not require specialized MS protocols but consistent, respectful, and structured care. Core actions include integrating sexual orientation and gender identity fields, preferred name/pronouns, and an organ inventory into the medical record; asking open questions about partners, living situation, and safety; routine screening for depression, anxiety, substance use, and sexual health with early referral; visible non-discrimination policies; gender-neutral forms and facilities; staff training in LGBTQ + cultural competence; and facilitation of LGBTQ+-affirming MS peer support (including virtual options) [328]. Disease-modifying therapies should be selected by standard clinical criteria rather than identity; for those initiating or modifying GAHT, clinicians should set expectations and plan surveillance proactively [331]. In sum, a biologically informed and culturally competent model—aligning GAHT and MS management, reducing avoidable delays and miscommunication, and providing affirming environments and support networks—offers a clear path to safer, more equitable outcomes for LGBTQ + people living with MS.

## Conclusions

Sex and gender are key determinants of interindividual differences in the risk of progression, clinical presentation, neuropathology, treatment response and access to care of the most common neurological disorders, and need to be considered in clinical practice. Further studies exploring sex differences in pathophysiology, risk factors, diagnostic and prognostic biomarkers of the most common neurological diseases are needed.

Gender effect on response to available treatments would deserve post-hoc analyses of clinical trials and real-world population studies to build a common knowledge of sex differences in efficacy and safety.

## Recommendations and future directions

Based on available evidence is it possible to formulate the following general recommendations:

- Consider sex-specific modifiable risk factors for the main neurological disorders (e.g. stroke, dementia, migraine etc.) in order to reduce the risk of diseases.
- Consider sex differences across the lifespan, including childhood, adolescence, and older age.
- Hormonal status may influence not just the risk, but also disease progression, clinical presentation and treatment response of common neurological disorders. It is important to consider the neurological disorders across the women’s lifespan (fertile life, pregnancy and postpartum health, aging and menopause).
- Consider the different clinical presentation and disease progression according to gender (i.e. Parkinson’s disease, Multiple Sclerosis), and act toward better diagnosis and prevention of complications.
- Consider a gender-oriented treatment taking into account possible differences in terms of efficacy, side effects, and possible effects on pregnancy and breastfeeding.
- Consider a tailored, sex-informed approach to deliver individualized care to patients with neurological disorders.
- Despite recognizing the importance of sex inclusiveness in clinical research, sex bias remains prevalent in biomedical research. It’s important to promote development of comprehensive and rigorous guidelines to ensure that sex and gender are appropriately integrated into the analysis and reporting of clinical research, as well as in the diagnosis and treatment of neurological disorders.
- Sustain public health interventions targeting modifiable risk factors, that offer a promising avenue for lowering disease prevalence.
- Despite several initiatives have been developed to create equal representation of men and women participants in biomedical research studies, women are still under-represented in clinical trials leading to consequences on the external validity of the benefit/risk assessments of launched drugs. Drug developers should consider including sex in clinical trial design, ensuring a proper stratification of men and women into experimental groups and pre-specified ad-hoc analyses of sex subgroups for both efficacy and safety of the drug under development.
- Promote research on the safety profiles of newer drugs during pregnancy to minimize the fetal risks.
- Research should extend beyond pregnancy and menstruation to investigate sex differences across the lifespan, including childhood, adolescence, and older age.
- Compared to men, women affected by neurological disorders often experience delays in receiving diagnoses and are more likely to undergo inappropriate interventions. Dedicated awareness campaigns for women are needed to guarantee equity of access to care.
- Identify the factors that cause delayed diagnosis for women in certain neurological conditions (i.e. stroke) or men (i.e. migraine) and address them appropriately in order to give better medical care and improve quality of life of patients.
- Promote studies to better characterize differences in the epidemiology, pathophysiology, clinical course, and management of neurological diseases among SGM populations, identify specific clinical needs and guide preventive and therapeutic strategies.
